# Assessing the consistency of CT-based ventilation imaging under noise reduction processing with simulated quantum noise using a nonrigid alveoli phantom

**DOI:** 10.3389/fradi.2025.1567267

**Published:** 2025-07-09

**Authors:** Shin Miyakawa, Hiraku Fuse, Kenji Yasue, Norikazu Koori, Masato Takahashi, Hiroki Nosaka, Shunsuke Moriya, Fumihiro Tomita, Tatsuya Fujisaki

**Affiliations:** ^1^ Department of Radiological Sciences, Ibaraki Prefectural University of Health Sciences, Ibaraki, Japan; ^2^ Department of Radiological Technology, Niigata University of Health and Welfare, Niigata, Japan; ^3^ Institute of Medicine, University of Tsukuba, Ibaraki, Japan; ^4^ Department of Radiation Oncology, St. Luke’s International Hospital, Tokyo, Japan

**Keywords:** computed tomography-based ventilation image, deformable image registration, noise reduction, nonrigid alveoli phantom, radiotherapy

## Abstract

**Background:**

Previous studies have reported that quantum noise inherently present in CT images hinders the generation of CT-based ventilation image (CTVI), while quantum noise reduction approaches that do not affect CTVI have not yet been reported.

**Aims:**

The purpose of this study was to evaluate the impact of noise reduction preprocessing on the accuracy and robustness of CTVI in relation to quantum noise present in CT images.

**Methods and material:**

To reproduce the quantum noise, Gaussian noise (SD: 30, 80, 150 HU) was added to each inhalation and exhalation CT image. CTVI_ref_ and CTVI_noise_ was generated from CT_ref_ and CT_noise_. A median filter and the noise reduction by the CNN were also applied to the CT image, which contained the quantum noise, and CTVI_med_ and CTVI_cnn_ was created in the same manner as CTVI_ref_. We evaluated whether the regions classified as high, middle, or low in CTVI_ref_ were accurately represented as high, middle, or low in CTVI_noise_, CTVI_med_ and CTVI_cnn_. Additionally, to evaluate the ventilation function of each voxel, we compared two-dimensional histograms of CTVI_ref_, CTVI_noise_, CTVI_med_ and CTVI_cnn_.

**Statistical analysis used:**

Cohen's kappa coefficient and Spearman's correlation were used to assess the agreement between CTVI_ref_ and each of the following: CTVI_noise_, CTVI_med_, and CTVI_cnn_.

**Results:**

CTVI_cnn_ significantly improved categorical consistency and voxel-level correlation of CTVI, particularly under high-noise conditions (150 HU), outperforming both CTVI_noise_ and CTVI_med_.

**Conclusions:**

CNN-based denoising effectively improved the accuracy and robustness of CTVI under quantum noise.

## 1 Introduction

A variety of imaging modalities exist to assess pulmonary ventilation. Examples include computed tomography (CT), dual-energy CT, magnetic resonance imaging, single-photon emission computed tomography (SPECT), and positron emission tomography (PET). These techniques can accurately assess the three-dimensional (3D) distribution of ventilatory function in a patient's lungs (1–3). In radiation therapy, treatment plans that utilize routine CT imaging and deformable image registration (DIR) to generate CT-based ventilation images (CTVIs) have demonstrated the ability to avoid areas of high ventilatory function within the lungs (4). The approach aims to reduce the dose administered to regions with elevated ventilatory function, creating a more targeted and personalized treatment strategy. This approach not only enhances the accuracy of predicting adverse lung events but also contributes to a more effective reduction in the occurrence of such events during treatment (5, 6). CT scans are used as part of routine radiation treatment procedures for most lung cancer patients and can provide additional functional information about the patient without requiring additional functional imaging equipment or methods. Treatment planning with CTVI is a practical, high-resolution, cost-effective, and time-saving approach that can be performed based on four-dimensional (4D) or expiratory and inspiratory CT images (4–9).

Studies are currently underway to validate the accuracy of CTVI. Radionuclide imaging is widely used to assess pulmonary function and is considered the standard of choice for assessing other functional imaging modalities (7, 8). Recent studies have demonstrated that CT-based and SPECT ventilatory function imaging have good spatial measurement accuracy and correlation (9, 10). In addition, clinical trials demonstrate that radiotherapy using CTVI significantly reduces dose to ventilated lung regions (NCT02528942, NCT02308709, NCT02843568) (11).

Accurate assessment of pulmonary ventilation function is crucial for using CTVI in treatment planning. Small changes in DIR parameters have been reported to cause large relative changes in the CTVI (12). The study noted that DIR-based images may not show accurate ventilatory function even when the spatial accuracy of the deformations is acceptable using target registration error (TRE). The quantum noise in CT images does not significantly affect the accuracy of DIR but may hinder the generation of accurate CTVI (13). A nonrigid alveoli phantom was developed to evaluate the CTVI, based on the assumption that an accuracy validation phantom is required to investigate the causes of these obstacles and improve CTVI accuracy (14). However, various problems related to CTVI methods have not yet been solved. It has been demonstrated that there is a significant difference in the CTVI produced via DIR when different DIR parameters are used, even after meeting the tolerance for DIR accuracy with this phantom (15).

CT images inevitably contain quantum noise, owing to the nature of x-ray images. It is desirable to use high-resolution CT images to create the CTVI. CTVI is used for both treatment planning and tracking pulmonary ventilation function using cone-beam CT acquired during treatment. Therefore, the accuracy of the CTVI must be independent of the CT image quality. To achieve this, the image quality must be improved using noise reduction and image correction techniques. Consequently, it could improve the accuracy of treatment planning and patient outcomes. Therefore, CTVI plays a crucial role in radiotherapy treatment planning, and it is desirable to improve its reliability and robustness through various methods.

In recent years, in addition to conventional filtering techniques such as median and Gaussian filters (16), image denoising methods using artificial intelligence (AI) have also been increasingly utilized in the field of medical imaging. In particular, deep learning methods based on convolutional neural networks (CNNs) have attracted attention as they can suppress noise while preserving structural details (17–19). Such AI-based preprocessing techniques are being explored as potential means to enhance the robustness and reproducibility of CT-based functional imaging, including CTVI.

In this study, we used a nonrigid alveoli phantom with ventilation functionality, which we developed as the world's first quality control tool for CTVI. We investigated the effect of preprocessing using both a conventional median filter and a deep learning-based denoising model on the accuracy and robustness of CTVIs. The purpose of this study is to evaluate how preprocessing methods, including AI-based denoising, affect the quality of CTVI, and to clarify their potential to improve robustness and accuracy in clinical applications.

## 2 Subjects and methods

### 2.1 CT datasets

The expiratory and inspiratory CT images were acquired using a 16-row detector CT scanner (Aquilion LB, Toshiba Medical Systems, Otawara, Japan). Image resolution was set to 0.78 × 0.78 × 3 mm, and a helical scan protocol was used. The scan parameters were set to 120 kVp, 300 mA, rotation time of 0.5 s, and slice thickness of 3.0 mm. The nonrigid alveoli phantom comprised an acrylic cylinder filled with polyurethane foam simulating alveoli, a polyurethane membrane simulating the diaphragm, a metal rod with piston function simulating respiratory muscles, and a polyurethane tube simulating the airway (14). Various motion patterns can be programmed to simulate breathing patterns of various frequencies. Additionally, airflow can be controlled by pressure changes in the vessel owing to diaphragm movement. The phantom was placed horizontally and adjusted to align with the longitudinal axis of the CT system. The respiratory cycle of the phantom was set to 10 s. The normal respiratory cycle is approximately 4 s; however, to focus only on quantum noise, the respiratory cycle of the phantom was set at which the motion artifact was as small as possible.

### 2.2 Simulation of quantum noise and noise reduction by the median filter

**Figure 1:**
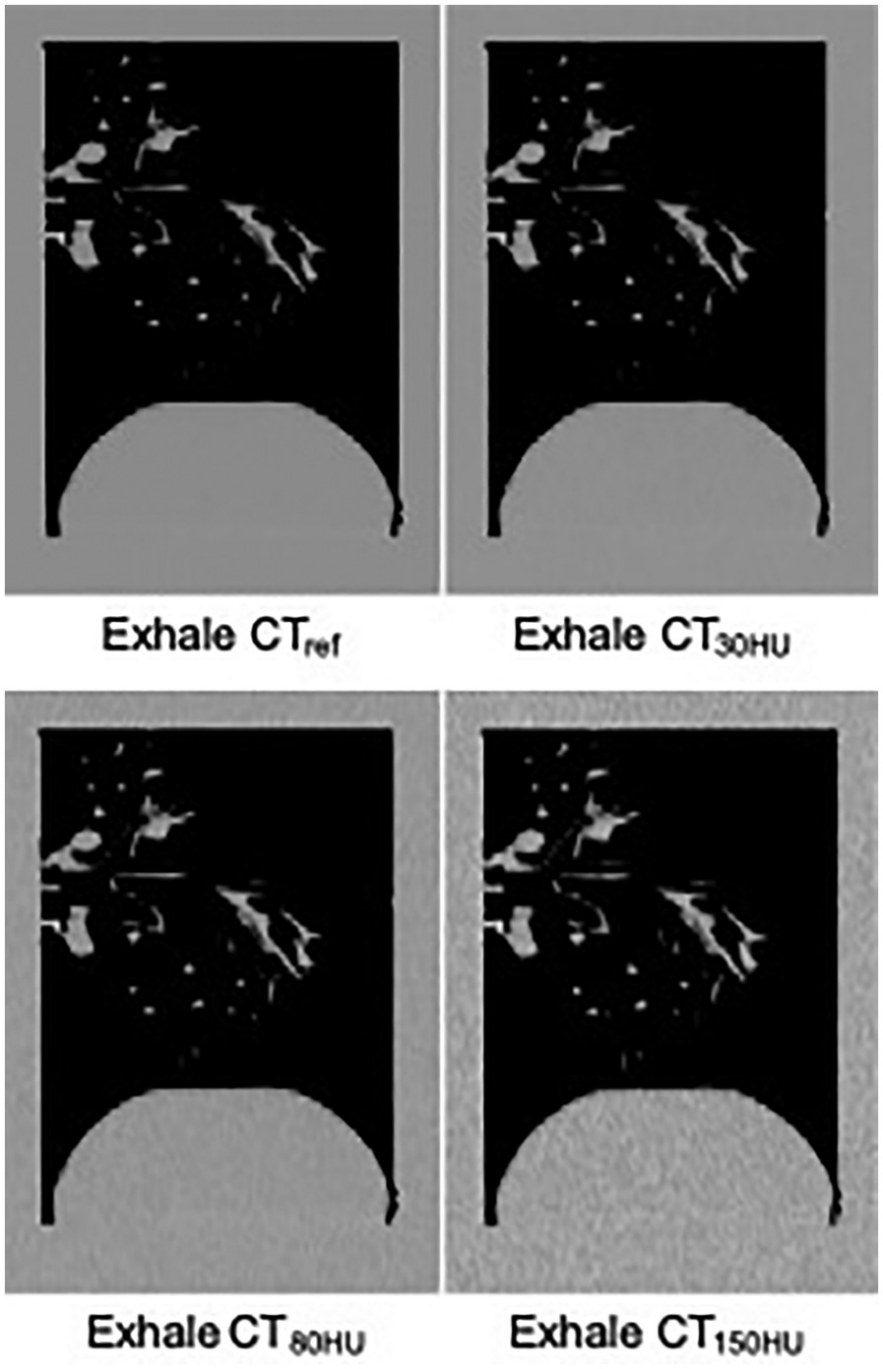
Comparison of exhale CT images simulating different noise levels.

Additional noise was applied to the CT images to simulate the quantum noise in a simplified manner. The amplitude of quantum noise can be mathematically approximated by a Gaussian distribution (20). In this study, a Python script was developed to add noise with varying standard deviation [0–150 Hounsfield units (HU)] based on a normal distribution. Quantum noise was added using this script to a set of three pairs of expiratory and inspiratory images (30, 80, and 150 HU) to simulate the quantum noise in the CT images, as shown in Figure 1. We developed a script to calculate the median filter for the CT images with simulated quantum noise and fit it to all simulated noise. The filter used a kernel size of 3 × 3.

### 2.3 Noise reduction by the deep learning-based denoising model

**Figure 2:**
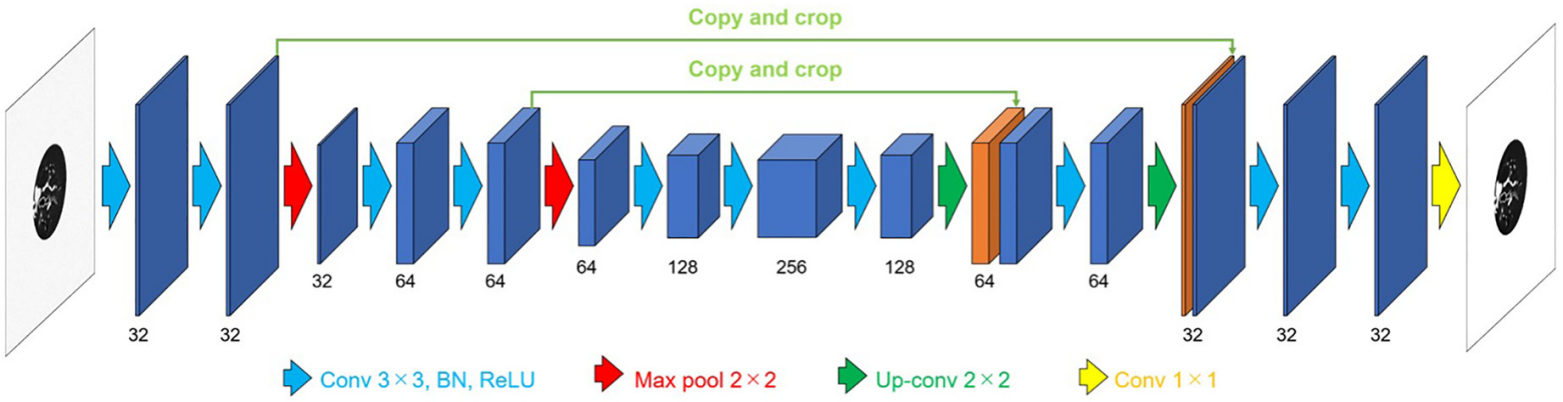
The denoising model based on a two-dimensional U-Net architecture.

We constructed a denoising model based on a two-dimensional U-Net architecture (24) (Figure 2), with a network depth of 3 and an initial number of filters set to 32. The input to the model was a noisy image, and the output was the corresponding denoised image. To enable fair comparison with conventional filtering methods, no normalization was applied to the pixel values. Each image had a resolution of 512 × 512 pixels, and 131 slices were used per subject. For training, 14 types of Gaussian noise with standard deviations of 10, 20, 50, 60, 70, 100, 110, 120, 130, 140, 170, 180, 190, and 200 HU were added to clean images. For validation, noise levels of 40, 90, and 160 HU were used, and for testing, levels of 30, 80, and 150 HU were selected. The model was trained using the Adam optimizer with a batch size of 8 for up to 500 epochs. The L1 norm loss is defined as shown in Equation 1:

(1) $$l(x,y)=\frac{1}{N}\overset{N}{\underset{n=1}{\sum }}\;|x_{n}-y_{n}|,\;$$

where $x_{n}$ and $y_{n}$ denote the predicted and ground-truth pixel values, respectively, and N is the total number of pixels. For the validation dataset, the clean images were used as the ground truth, and the model yielding the lowest loss between the output and the clean images was selected for final testing. All training and evaluation were performed on a workstation equipped with an Intel Core i9-9920X 3.5 GHz twelve-core processor, 32 GB RAM, and an NVIDIA GeForce RTX 2080 Ti GPU running Ubuntu 22.04.4 LTS with NVIDIA Driver 535.183.01, CUDA 12.1, and cuDNN 8.9.7.29-1.

### 2.4 Deformable image registration

In this study, the inhalation image was deformed to match the reference expiration image. Deformations were performed on a set of ten pairs of expiratory and inspiratory images: the reference image CT_ref_ without additional noise, CT_noise_ with noise (30, 80, and 150 HU), and CT_med_ and CT_cnn_, which are denoised versions of CT_noise_ using a median filter and the CNN model, respectively. Deformable image registration was performed using NiftyReg (version 1.4.2), a free and open-source software package for non-rigid image registration. NiftyReg uses a B-spline-based free-form deformation algorithm, which estimates the transformation between moving and reference images by optimizing a normalized mutual information while applying smoothness constraints. The DIR parameters used for these deformations were the optimal parameters reported in a previous study (15): “*bending-energy penalty term*,” introduced in the cost function to smooth deformations; “*max number of iterations*,” which affects the computation time; “*number of levels to perform*,” which refers to the number of optimization calculations; and “*Jacobian-based penalty term,*” which penalizes large local volume changes and prevents folding (21). The deformation was performed in four steps, following the deformation strategy previously reported as optimal in earlier studies (15). Each step was visually checked, and if the deformation was over-deformed, the deformation step was omitted. The deformation vector field was obtained at each step. It was input at the next step and integrated for each deformation. The CT scans in this study were performed in one imaging session and the phantom outline was not moving; therefore, no rigid registration was performed before the deformation process.

### 2.5 CT-based ventilation imaging

The sum of the deformation vector field acquired for each of the seven paired sets was converted to a Jacobian determinant to obtain the respective CTVI_ref_, CTVI_noise_ (30, 80, and 150 HU), and CTVI_med_ (30, 80, and 150 HU), and CTVI_cnn_ (30, 80, and 150 HU). The DIR-based Jacobian metric was developed by Reinhardt et al. and is a measure of spatial volume change; it ensures that local volume changes do not alter the signal throughout the volume (22). The Jacobian determinant was calculated for each voxel in the phantom using (Equation 2).

(2) $$\begin{array}{r} \begin{array}{c} \mathrm{Jacobian}\;\mathrm{determinant}(x,y,z)\\ =\left|\begin{array}{ccc} 1+\frac{\partial u_{x}(x,y,z)}{\partial x} & \frac{\partial u_{x}(x,y,z)}{\partial y} & \frac{\partial u_{x}(x,y,z)}{\partial z}\\ \frac{\partial u_{y}(x,y,z)}{\partial x} & 1+\frac{\partial u_{y}(x,y,z)}{\partial y} & \frac{\partial u_{y}(x,y,z)}{\partial z}\\ \frac{\partial u_{z}(x,y,z)}{\partial x} & \frac{\partial u_{z}(x,y,z)}{\partial y} & 1+\frac{\partial u_{z}(x,y,z)}{\partial z} \end{array}\right| \end{array} \end{array}$$

where u_x_, u_y_, and u_z_ are the x, y, and z components of u, respectively. Jacobian determinant measures the expansion and contraction at position (x, y, z) in the image. When Jacobian determinant is greater than one, local tissue expansion is present, and when Jacobian determinant is less than one, local tissue contraction is present. Jacobian determinant is a relative measure of ventilatory functionality on a voxel-by-voxel basis within the lung.

### 2.6 Evaluation of spatial deformation accuracy by DIR

**Figure 3:**
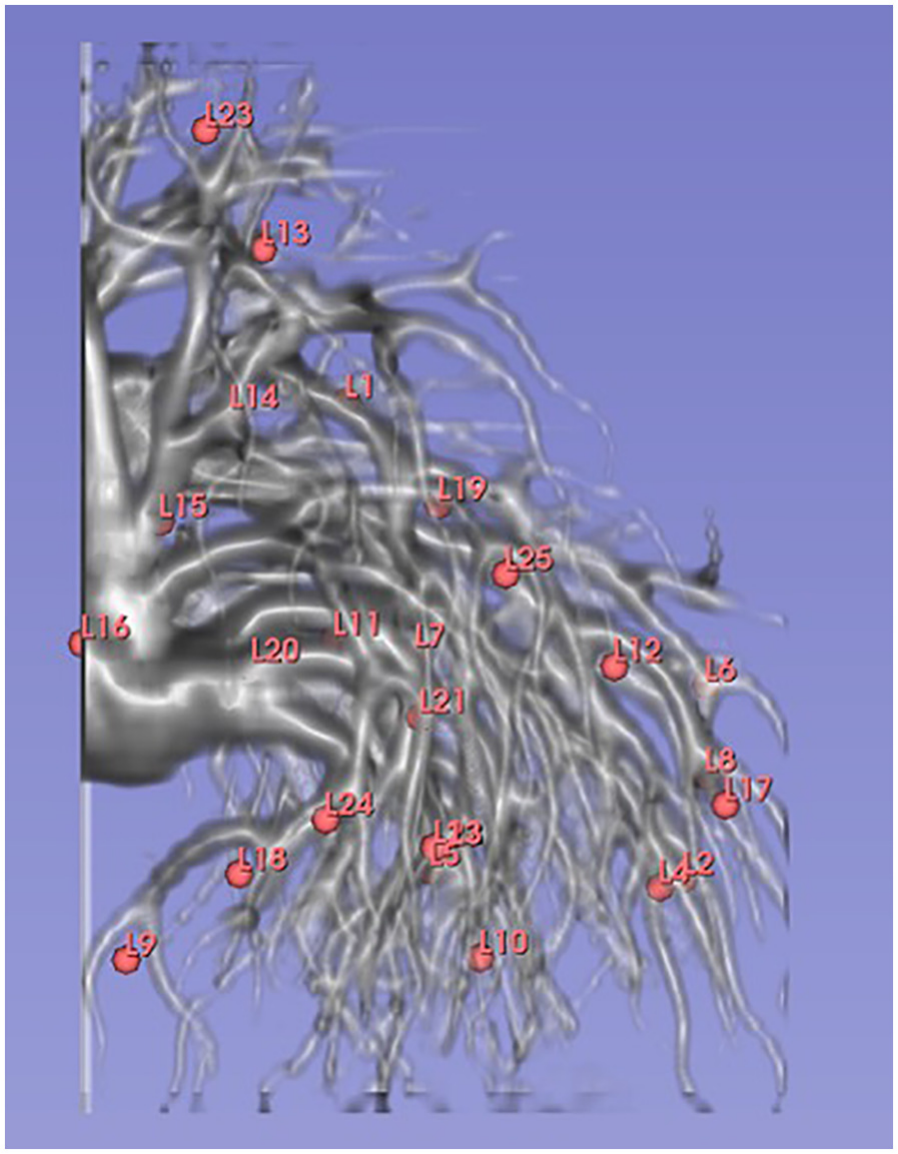
Twenty-five landmark setups placed in a volume near the pulmonary vessels and bronchi in a nonrigid alveoli phantom.

Twenty-five landmarks were manually placed by an experienced medical physicist in a volume near the pulmonary vessels and bronchi in a nonrigid alveoli phantom (Figure 3). The target displacement error, that is, the displacement of a landmark due to respiratory motion, was measured as the Euclidean distance between the exhalation and inhalation images. The Euclidean distance was calculated using the formula shown in Equation 3:

(3) $$\begin{array}{c} \sqrt{{\left(x_{r}-x_{t}\right)}^{2}+{\left(y_{r}-y_{t}\right)}^{2}+{\left(z_{r}-z_{t}\right)}^{2}}, \end{array}$$

where (x_r_, y_r_, z_r_) and (x_t_, y_t_, z_t_) are the landmark coordinates of the reference and target images, respectively. To evaluate the spatial accuracy of DIR with added noise, we used the Euclidean distance between the corresponding landmarks defined in the expiratory and deformed inspiratory images to calculate the Euclidean distance, which is denoted as TRE. TRE represents the spatial 3D distance discrepancy. When the deformed image perfectly matches the reference expiratory image (Euclidean distance = 0), TRE is equal to zero. Relative spatial accuracy was evaluated and compared with the spatial accuracy of the reference noiseless DIR.

### 2.7 Global consistency analysis using kappa statistics

To evaluate the clinical consistency of CTVI for treatment planning, each voxel in the CTVI was categorized into three regions—high, middle, and low ventilation—by evenly dividing the range of ventilation values in CTVI_ref_. This categorization reflects a typical clinical scenario where high-ventilation regions are avoided during irradiation. The same classification thresholds were applied to all other CTVIs, including CTVI_noise_ (30, 80, and 150 HU), CTVI_med_ (30, 80, and 150 HU), and CTVI_cnn_ (30, 80, and 150 HU). Although the absolute ventilation values may differ, consistency was defined as the regions categorized as high, middle, or low in CTVI_ref_ being similarly categorized in the compared CTVIs. To quantify consistency, the proportion of voxels in each test CTVI that retained the same categorical label (high, middle, or low) as in CTVI_ref_ was calculated. The degree of agreement was assessed using Cohen's kappa coefficient.

### 2.8 Voxel-based local evaluation using 2D histograms and spearman correlation

To evaluate the consistency of local ventilation function in each voxel, a two-dimensional (2D) histogram was constructed by plotting the Jacobian determinant value of each voxel in CTVIref against the corresponding value in CTVI_noise_ (30, 80, and 150 HU). Similarly, 2D histograms were created for CTVI_med_ (30, 80, and 150 HU) and CTVI_cnn_ (30, 80, and 150 HU), which were generated by denoising CT_noise_ using a median filter and the CNN model, respectively. All histograms were generated based on voxel-wise spatial correspondence with CTVI_ref_, enabling direct comparison of local ventilation values. Spearman's rank correlation coefficients were calculated from the 2D histograms to evaluate the consistency between each CTVI and the reference.

## 3 Results

### 3.1 Evaluation of DIR spatial deformation accuracy

**Figure 4:**
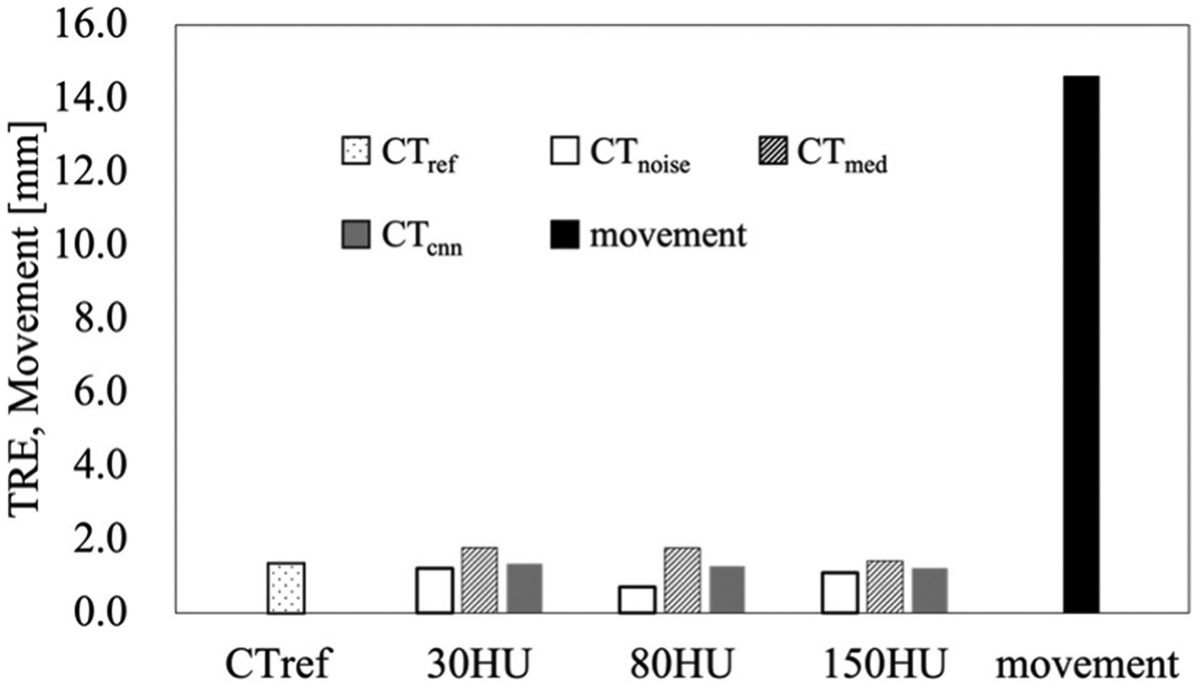
Comparison of TRE values was performed for cT_noise_, cT_med_, cT_cnn_, and cT_ref_ at different noise levels (30, 80, and 150 HU), where cT_ref_ represents the DIR results based on CT images without added quantum noise. In this context, movement indicates the displacement caused by respiratory motion between the expiratory and inspiratory phases.

Figure 4 presents a comparison of TRE values, indicating the spatial accuracy of DIR between CT images containing noise and CT image pairs with noise removed using the median filter and CNN-based denoising. The average displacement between the expiratory and inspiratory CT images was 14.59 ± 6.42 mm. The mean TRE values of the 25 landmarks were 1.39 ± 0.89 mm (maximum 2.95 mm) for CT_ref_. The mean TRE values of the 25 landmarks were 1.22 ± 0.65 mm (maximum 2.54 mm), 0.71 ± 0.45 mm (maximum 1.89 mm), and 1.10 ± 0.83 mm (maximum 2.71 mm) for CT_noise_ (30, 80, and 150 HU). The mean TRE values for CT_med_ were 1.78 ± 0.71 mm (maximum 2.93 mm), 1.77 ± 0.78 mm (maximum 2.94 mm), and 1.42 ± 0.65 mm (maximum 2.80 mm) at 30, 80, and 150 HU, respectively. The mean TRE values for CT_cnn_ were 1.34 ± 0.00 mm (maximum 2.55 mm), 1.28 ± 0.00 mm (maximum 2.82 mm), and 1.22 ± 0.00 mm (maximum 2.45 mm) at 30, 80, and 150 HU, respectively. TREs for all conditions, including noisy and denoised images, remained within 3 mm. When comparing mean TRE values between CT_noise_ and denoised images at each noise level, TREs were higher in CT_med_, while CT_cnn_ yielded values similar to or slightly higher than CT_noise_.

### 3.2 Evaluation of CTVIs

#### 3.2.1 Visual assessment of CTVIs

**Figure 5:**
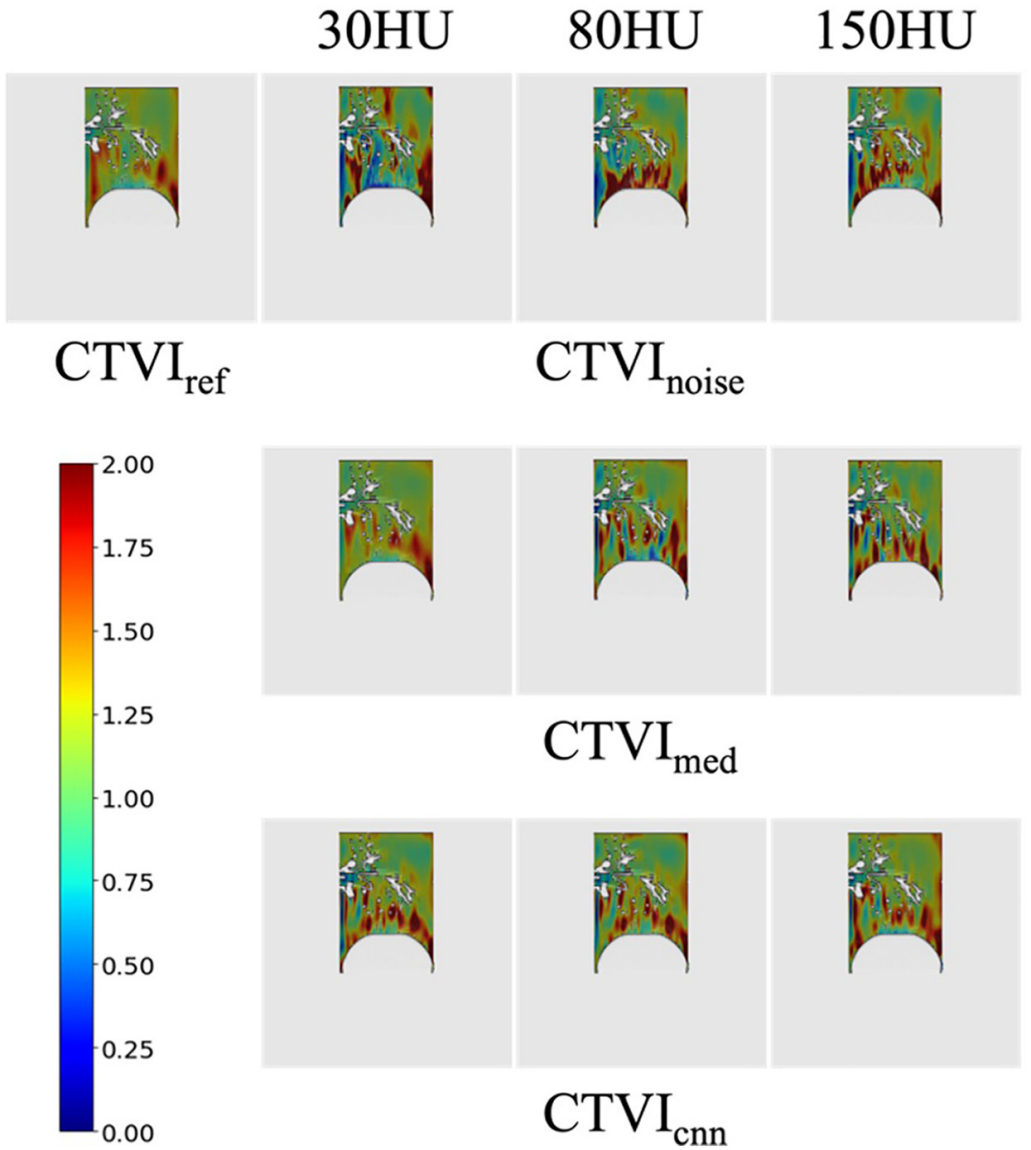
CTVI_noise_, CTVI_med_ and CTVI_cnn_ at different noise levels. High-functioning regions are shown in red, while regions with decreased lung ventilation are displayed in blue.

Figure 5 shows a visual comparison of CTVIs, including CTVI_ref_, CTVI_noise_, CTVI_med_, and CTVIcnn at different noise levels. The visual assessment reveals that, compared to CTVI_ref_, the location of high-functioning regions near the diaphragm remains consistent. However, additional high-functioning regions not observed in CTVI_ref_ are present, and the resolution of ventilatory function distribution is reduced. The visual assessment indicates that CTVI_med_ (30 HU) is closer to CTVI_ref_ than the corresponding CTVI_noise_ shown in Figure 5, suggesting that noise reduction improves the visual accuracy of CTVI under lower noise conditions. CTVI_cnn_ appear visually closer to CTVI_ref_ than CTVI_noise_ at all noise levels, indicating that noise reduction improves the visual accuracy of CTVI. Among the three noise levels, CTVI_cnn_ at 150 HU shows a particularly notable improvement over CTVI_noise_, suggesting that CNN-based denoising enhances the visualization of ventilation distribution under high noise conditions.

#### 3.2.2 Global consistency analysis using kappa statistics

**Figure 6:**
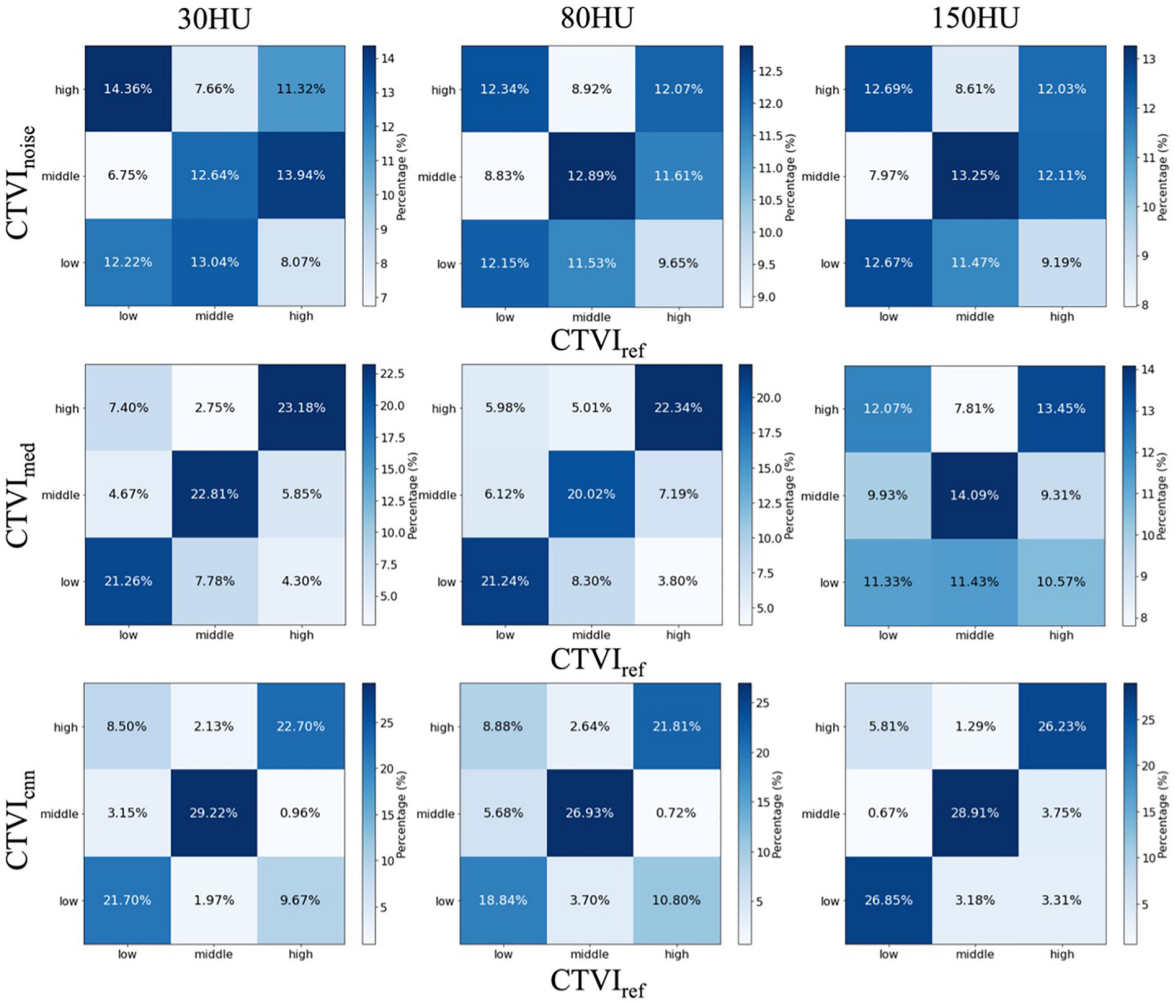
Proportion of categorized voxels across high, middle, and low ventilation regions for CTVI_noise_, CTVI_med_ and CTVI_cnn_ compared to CTVI_ref_. The horizontal axis represents CTVI_ref_, while the vertical axis shows the three ventilation categories (high, middle, and low) for CTVI_noise_, CTVI_med_ and CTVI_cnn_. Darker colors indicate higher agreement in classification.

To evaluate the clinical utility of CTVI for treatment planning, ventilation values in CTVI_ref_ were evenly divided into three regions: high, middle, and low ventilation. The same categorization was applied to CTVI_noise_, CTVI_med_ and CTVI_cnn_ at each noise level. Consistency was defined as regions categorized as high, middle, or low in CTVI_ref_ being similarly categorized in CTVI_noise_, CTVI_med_ and CTVI_cnn_, regardless of absolute ventilation values. If the categorizations were perfectly consistent, the percentage of voxels correctly matching high, middle, and low regions would be 33.3% for each category. Figure 6 summarizes the percentage of voxels that correctly matched high, middle, and low regions between CTVI_ref_ and CTVI_noise_. For example, at 30 HU noise, only 11.32%, 12.64%, and 12.22% of voxels in the high, middle, and low regions of CTVI_ref_ were correctly identified in CTVI_noise_, respectively, with notable mismatches such as 14.36% of voxels categorized as low in CTVI_ref_ being misclassified as high in CTVI_noise_. In contrast, CTVI_med_ at 30 HU showed substantially improved agreement, with 21.26%, 22.81%, and 23.18% correctly matching the high, middle, and low regions of CTVI_ref_, respectively. Additionally, at 80 HU noise, CTVI_med_ also demonstrated an improvement in consistency compared to CTVI_noise_. The percentages of voxels correctly categorized as high, middle, and low in CTVI_med_ were 21.24%, 20.02%, and 22.34%, respectively, indicating a better agreement with CTVI_ref_ compared to CTVI_noise_, where the corresponding percentages were only 12.15%, 12.89%, and 12.07%. This result highlights that the noise reduction processing improved clinical consistency not only at 30 HU but also at 80 HU noise levels. In addition, CTVI_cnn_ showed consistent improvement across all noise levels. Notably, at 150 HU, the consistency between CTVI_cnn_ and CTVI_ref_ was significantly higher than that of CTVI_noise_. The percentage of correctly matched voxels was 26.23%, 28.91%, and 26.85% for high, middle, and low ventilation regions, respectively, compared to only 12.03%, 13.25%, and 12.67% for CTVI_noise_. These results highlight that CNN-based denoising particularly improves clinical consistency under high-noise conditions. Cohen's kappa coefficients further quantified the agreement between CTVI_ref_ and both CTVI_noise_ and CTVI_med_. Table 1 presents these results, showing that kappa values for CTVI_noise_ were 0.043, 0.057, and 0.069 at noise levels of 30 HU, 80 HU, and 150 HU, respectively. For CTVI_med_, the kappa values were significantly higher at lower noise levels (0.51 at 30 HU and 0.45 at 80 HU), but the consistency diminished at 150 HU (0.083). CTVI_cnn_ exhibited consistently high kappa values across all noise levels, with 0.60 at 30 HU, 0.51 at 80 HU, and 0.73 at 150 HU, indicating superior categorical agreement with CTVI_ref_ compared to both CTVI_noise_ and CTVI_med_. These results support the effectiveness of CNN-based denoising in preserving clinically relevant ventilation patterns, even under high noise conditions.

**Table 1:** Cohen's kappa coefficients of CTVIs for CTVI_ref_.

CTVI methods	30 HU (*P* value)	80 HU (*P* value)	150 HU (*P* value)
CTVI_noise_	0.043 (<.0001)	0.057 (<.0001)	0.069 (<.0001)
CTVI_med_	0.51 (<.0001)	0.45 (<.0001)	0.083 (<.0001)
CTVI_cnn_	0.60(<.0001)	0.51 (<.0001)	0.73 (<.0001)

#### 3.2.3 Voxel-based local evaluation using 2D histograms and spearman correlation

**Figure 7:**
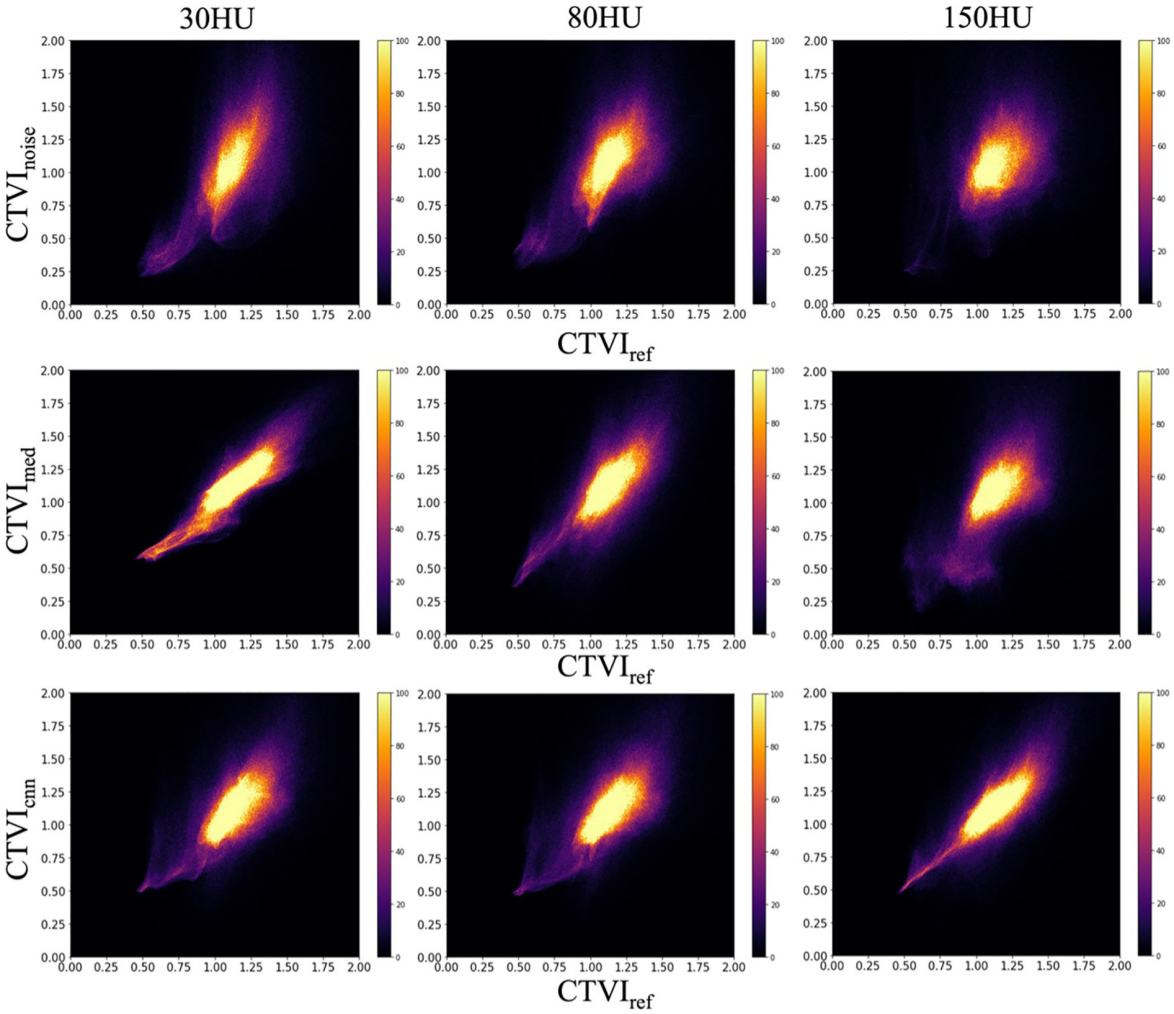
Two-dimensional histograms of CTVI_noise_, CTVI_med_ and CTVI_cnn_ at different noise levels with respect to CTVI_ref_. The horizontal axis represents CTVI_ref_, while the vertical axis shows the voxel-based lung ventilation of CTVI_noise_, CTVI_med_ and CTVI_cnn_. Perfect agreement is indicated by the histogram distribution along y = x.

To assess the consistency of local ventilation function, a voxel-by-voxel comparison between CTVI_ref_ and both CTVI_noise_, CTVI_med_ and CTVI_cnn_ was conducted using 2D histograms (Figure 7). These histograms demonstrate that the distribution improves as it approaches y = x, indicating greater consistency with CTVI_ref_. At a noise level of 30 HU, the histogram of CTVI_med_ shows a marked improvement in consistency compared to CTVI_noise_, as the distribution is more concentrated along y = x. However, at noise levels of 80 and 150 HU, no significant improvement was observed. In contrast, CTVI_cnn_ demonstrated a different trend: while there was no notable improvement in the 2D histograms at 30 and 80 HU, the histogram at 150 HU showed greater alignment with the y = x line, suggesting improved voxel-level consistency. Table 2 presents the Spearman correlation coefficients for these comparisons. The results show that CTVI_med_ exhibits better correlation with CTVI_ref_ than CTVI_noise_ at lower noise levels, but the correlation decreases as the noise level increases. Despite this, a statistically significant trend toward improvement is observed (<.0001). The Spearman correlation coefficients for CTVI_cnn_ were 0.59, 0.65, and 0.83 at 30, 80, and 150 HU, respectively. This indicates that although CTVI_cnn_ did not improve correlation at lower noise levels, it showed a substantial improvement at 150 HU (<.0001).

**Table 2:** Spearman correlation coefficients of CTVIs for CTVI_ref_.

CTVI methods	30 HU (*P* value)	80 HU (*P* value)	150 HU (*P* value)
CTVI_noise_	0.61 (<.0001)	0.59 (<.0001)	0.33 (<.0001)
CTVI_med_	0.87 (<.0001)	0.70 (<.0001)	0.61 (<.0001)
CTVI_cnn_	0.59 (<.0001)	0.65 (<.0001)	0.83 (<.0001)

## 4 Discussion

Despite the extensive body of research on CTVI (4–10), few studies have employed phantoms capable of replicating human-like ventilation in the context of clinical applications. While it is well known that noise significantly affects CT image analysis, the impact of noise reduction preprocessing on the accuracy and consistency of CTVI remains insufficiently explored. In this study, quantum noise was simulated in CT images using a nonrigid alveoli phantom designed to mimic lung motion.

In this study, the spatial accuracy of the DIR was evaluated by simulating noise levels from 0 to 150 HU. For all CT_ref_, CT_noise_, CT_med_ and CT_cnn_, the deformation accuracy was within 3 mm of the tolerances given in TG-132 (23), regardless of the noise level. Although previous studies have investigated noise levels of 200 HU (13), in the initial experiments of this study, the noise level of 200 HU resulted in over-deformation due to DIR for the same deformation parameters, and an accurate CTVI could not be established. This result suggests that noise levels above 200 HU significantly affect the deformation accuracy of the DIR. High noise levels may lead to errors in the DIR algorithm. Therefore, the results of this study suggest that the DIR technique has sufficient accuracy for generating CTVI at quantum noise levels up to 150 HU. Although all TRE values were within the TG-132 tolerance of 3 mm, CT_med_ and CT_cnn_ exhibited slightly higher TREs compared to CT_noise_. One possible explanation is that the denoising process may have smoothed out anatomical features critical for deformable registration, resulting in slightly reduced precision. Alternatively, mild quantum noise may have enhanced local contrast in CT_noise_, unintentionally aiding DIR alignment. However, these differences remained within the clinically acceptable margin and are unlikely to affect the final CTVI outcome.

Table 1; Figure 6 illustrate the impact of quantum noise and the application of preprocessing filters on the consistency of CTVI from a clinical perspective. When using CTVI for treatment planning, lung ventilation is categorized into three levels—high, middle, and low—and treatment plans are designed to avoid high-function regions. In CTVI_ref_, approximately 33% of the lung ventilation is classified into each category. Ideally, in cases of accurate classification, the relationships between CTVI_ref_, CTVI_noise_, CTVI_med_ and CTVI_cnn_ should result in high-high, middle-middle, and low-low matches approaching 33%. Focusing on CTVI_noise_, the maximum agreement across all noise levels was only 13.25%, indicating significant misclassification of lung ventilation when quantum noise is present. In contrast, CTVI_med_ achieved over 20% agreement in all ventilation categories at noise levels below 80 HU. A particularly important observation is the proportion of regions classified as high in CTVI_ref_ but misclassified as low. This proportion was kept below 4.3% at its maximum. In addition, CNN-based denoising further improved the consistency of CTVI at all noise levels. Notably, CTVI_cnn_ achieved the highest agreement with CTVI_ref_, particularly under high-noise conditions. At 150 HU, CNN showed the greatest improvement in both categorical agreement and voxel-wise correlation (*κ* = 0.73, Spearman *ρ* = 0.83), outperforming both CTVI_noise_ and CTVI_med_. These results indicate that CNN-based denoising has strong potential to enhance the robustness of CTVI, even under clinically challenging noise conditions.

Table 2; Figure 7 focus on the voxel-level accuracy of CTVI, demonstrating the local effects of quantum noise and the application of preprocessing filters on CTVI accuracy using two-dimensional histograms and Spearman correlation. The results of this study confirmed that as noise levels increase, the accuracy of CTVI decreases, proving that quantum noise is a significant factor that hinders the accuracy of CTVI. However, CNN-based denoising yielded stronger improvements in correlation, especially at 150 HU, suggesting it is a more robust solution in high-noise environments.

Interestingly, while Cohen's kappa coefficients and Spearman correlation coefficients generally showed consistent trends, some discrepancies were noted. For example, at 150 HU, CTVI_med_ yielded a relatively high Spearman correlation (*ρ* = 0.61) but a low kappa value (*κ* = 0.083), suggesting that voxel-wise rankings were preserved even though many values crossed categorical thresholds. Conversely, at 30 HU, CTVI_cnn_ showed a high kappa (*κ* = 0.60) despite having a lower Spearman correlation (*ρ* = 0.59), indicating that category-level agreement was strong, while voxel value variations limited rank correlation. These findings emphasize that categorical and continuous metrics capture different aspects of agreement, and highlight the need for using both to comprehensively evaluate CTVI accuracy.

Limitations of this study include the lack of comparison with vendor-provided denoising techniques and the absence of hybrid preprocessing strategies. Future work should explore combining CNN-based and conventional filtering approaches and testing these methods in patient datasets.

## 5 Conclusion

This study quantitatively evaluated the effect of preprocessing on the accuracy and robustness of the CTVI using a nonrigid alveoli phantom with ventilation functionality, developed as the world's first quality control tool for CTVI. We demonstrated that quantum noise significantly impairs the accuracy and consistency of CTVI. While median filtering was shown to be a simple and effective method for mitigating this effect, CNN-based denoising provided superior performance, particularly under high-noise conditions. These findings suggest that both conventional and AI-based preprocessing approaches contribute to improving the quality of CTVI, with deep learning methods offering strong potential to enhance robustness and accuracy in clinical applications.

## Data Availability

The original contributions presented in the study are included in the article/Supplementary Material, further inquiries can be directed to the corresponding author.
